# Possible Involvement of F1F0-ATP synthase and Intracellular ATP in Keratinocyte Differentiation in normal skin and skin lesions

**DOI:** 10.1038/srep42672

**Published:** 2017-02-17

**Authors:** Xie Xiaoyun, Han Chaofei, Zeng Weiqi, Chen Chen, Lu Lixia, Liu Queping, Peng Cong, Zhao Shuang, Su Juan, Chen Xiang

**Affiliations:** 1 Department of Dermatology, XiangYa Hospital, Central South University, Changsha, China; 2 Department of Rheumatology and Immunology, XiangYa Hospital, Central South University, Changsha, China; 3 Department of Plastic and Reconstructive Surgery, The Third XiangYa Hospital, Central South University, Changsha, China; 4 Hunan Key Laboratory of Skin Cancer and Psoriasis, XiangYa Hospital, Central South University, Changsha, China; 5 Department of Nephrology, XiangYa Hospital, Central South University, Changsha, China

## Abstract

The F1F0-ATP synthase, an enzyme complex, is mainly located on the mitochondrial inner membrane or sometimes cytomembrane to generate or hydrolyze ATP, play a role in cell proliferation. This study focused on the role of F1F0-ATP synthase in keratinocyte differentiation, and its relationship with intracellular and extracellular ATP (InATP and ExATP). The F1F0-ATP synthase β subunit (ATP5B) expression in various skin tissues and confluence-dependent HaCaT differentiation models was detected. ATP5B expression increased with keratinocyte and HaCaT cell differentiation in normal skin, some epidermis hyper-proliferative diseases, squamous cell carcinoma, and the HaCaT cell differentiation model. The impact of InATP and ExATP content on HaCaT differentiation was reflected by the expression of the differentiation marker involucrin. Inhibition of F1F0-ATP synthase blocked HaCaT cell differentiation, which was associated with a decrease of InATP content, but not with changes of ExATP. Our results revealed that F1F0-ATP synthase expression is associated with the process of keratinocyte differentiation which may possibly be related to InATP synthesis.

Differentiation is one of the most important physiological functions of keratinocytes for epidermal renewal and barrier formation. Various kinds of skin diseases are characterized by impairment of epidermal differentiation such as psoriasis, keratoacanthoma, verruca vulgaris, etc^1^,^2^. The whole differentiation process is tightly regulated, with certain genes being strictly expressed at specific moments in the whole process^3^,^4^, and some of them are widely used as differentiation markers. More specifically, expression of keratin markers K5 and K14 is restricted to the basal layer, while K1, K10, involucrin, and transglutaminase-1 indicate a differentiated phenotype^1^. Involucrin and transglutaminase-1 are both widely accepted markers in keratinocyte differentiation. They both play important roles in cornified envelope formation, and thus represent for an early stage of keratinocyte terminal differentiation. Involucrin is an early marker of terminal differentiation. As far as K10 is concerned, it is upregulated during the transition of the basal keratinocyte from the basal layer to the spinous layer to form intermediate filament and help compose the cell skeleton, thus widely applied as a marker to represent for keratinocyte early differentiation.

Recent studies have revealed a possible relationship among mitochondrial oxidative metabolism, keratinocyte differeantiation and skin carcinogenisis, in which the underlying mechanisms stay unclear^5^. As the end product of respiratory chain, ATP has been long involved in keratinocyte differentiation^5^. As far as InATP is concerned, research shows that a decrease of mitochondrial inner membrane potential and InATP production can trigger keratinocyte differentiation^5^. ExATP has recently been recognized as a messenger molecule for cell-cell communication, and can have broad spectrum of regulating effects in keratinocytes proliferation, differentiation, and cell death by binding specifically to membrane purinergic receptors^6^,^7^,^8^.

F1F0-ATP synthase is a widely expressed and evolutionarily conserved transmembrane protein complex that is mainly located on the mitochondrial inner-membrane, also known as the mitochondrial Complex V^9^. In most kinds of eukaryotic cells and tissues, it plays critical roles in cell energy metabolism and cellular homeostasis maintenance through ATP synthesis or hydrolysis coupled with H^+^ transport (passive or active), depending on the transmembrane proton gradient^9^,^10^. F1F0-ATP synthase is composed of 16–18 subunits, including α, β, γ, δ, ε and a, b, c, that respectively constitute two major sectors, the F1 and F0^11^. The β subunit (ATP5B) contains the ATP and ADP binding site (the catalytic site), which makes it the active center of the enzyme^12^,^13^.

The F1F0-ATP synthase was once thought to be restricted on mitochondrial inner membrane in eukaryotic cells, but recent studies have shown that it is also located on the cytoplasmic membrane of keratinocytes^14^, vascular endothelial cells^15^, adipocytes^16^, NSCLC cells^17^,^18^, and osteosarcoma cells^19^. In keratinocytes, F1F0-ATP synthase that is located on the cytoplasmic membrane is one of the main producers of ExATP^14^.

Numbers of small molecular inhibitors with different active sites and time phases are commonly used in the research of F1F0-ATP synthase functions^20^. Oligomycin, an antibiotic produced by Streptomyces diastatochromogens, can bind to the F0 sector, and inhibit the InATP synthesis and hydrolysis^21^; while another inhibitor, piceatannol, mainly inhibits the ExATP synthesis under normal curlture conditions by interacting with the F1 sector^22^,^23^. These inhibitors provide us ways to regulate the functioning status of F1F0-ATP synthase and thus the InATP and ExATP content, to explore its downstream effects.

Although at the center of ATP generation and cell energy metabolism, the relationship between F1F0-ATP synthase and other physiological and pathological behaviors (such as differentiation) has rarely been studied. Based on the important role that InATP and ExATP play during keratinocyte differentiation, the aim of the present study was to investigate whether F1F0-ATP synthase is directly related to this process and to explore the mechanisms in details. Results of the present study could help to determine new targets for the treatment of aberrant keratinocyte proliferation diseases.

## Results

### ATP5B expression is increased with epidermis differentiation in normal skin, some epidermis hyper-proliferative diseases, and SCC tissues

**Figure 1:**
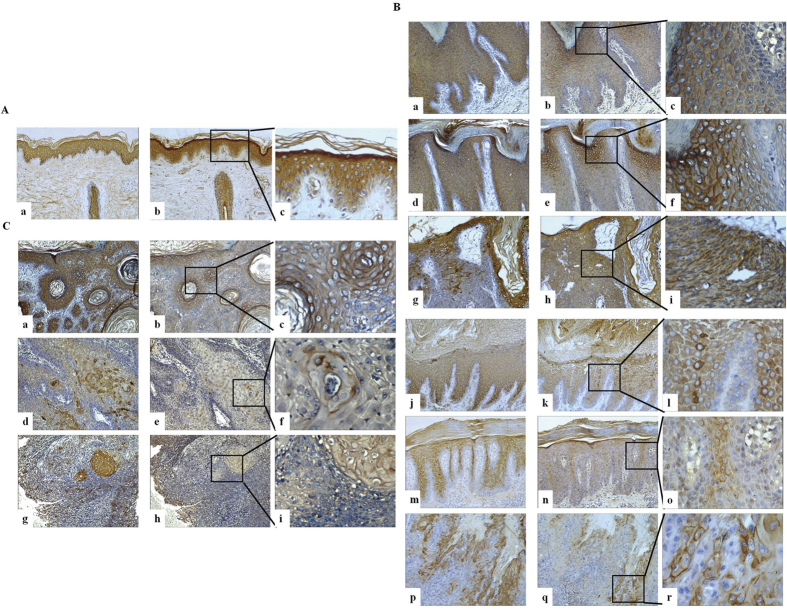
Differentiation-related ATP5B expression in normal skin and skin diseases. Expression of ATP5B and K10 in normal skin and skin diseases was assessed using IHC. K10 (lane 1, 100×) and ATP5B (lane 2, 100×, lane 3, 400×) expression in (**A**) normal skin, (**B**) chronic dermatitis (a–c), prurigo nodularis (d–f), seborrheic keratosis (g–i), verruca vulgaris (j–l), psoriasis (m–o), and keratoacanthoma (p–r), and in **(C)** well-differentiated SCC (a–c), moderately-differentiated SCC (d–f), and poorly-differentiated SCC (g–i).

Expression of ATP5B and K10 was analyzed by IHC in samples from nine normal skin, six chronic dermatitis, five prurigo nodularis, seven keratosis seborrheic, nine verruca vulgaris, 25 psoriasis, five keratoacanthoma, and nine SCC. K10, as an early marker of keratinocyte differentiation, was expressed in the suprabasal layers of normal epidermis. It slightly moved up to intermediate layers and periderm in epidermis hyper-proliferative skin diseases such as psoriasis. It was low or even absent in SCC tissues, especially those with poor differentiation status (Fig. 1).

ATP5B displayed a similar pattern of expression as K10 in normal epidermis and other skin diseases. To be specific, the expression of ATP5B was high in keratinocyte (or SCC cells) differentiation either in normal or pathologic conditions. Furthermore, ATB5B was strongly expressed in normal epidermis, and less expressed in other epidermis hyper-proliferative skin diseases and SCC (Fig. 1).

### F1F0-ATP synthase inhibitor oligomycin inhibits InATP synthesis and involucrin expression

Oligomycin and piceatannol are both F1F0-ATP synthase inhibitors with different active sites and time phases. Specifically, oligomycin mainly inhibits InATP synthesis while piceatannol mainly decreases ExATP content under normal culture conditions. The doses of oligomycin used in the present study were based on previous studies^24^,^25^

**Figure 2:**
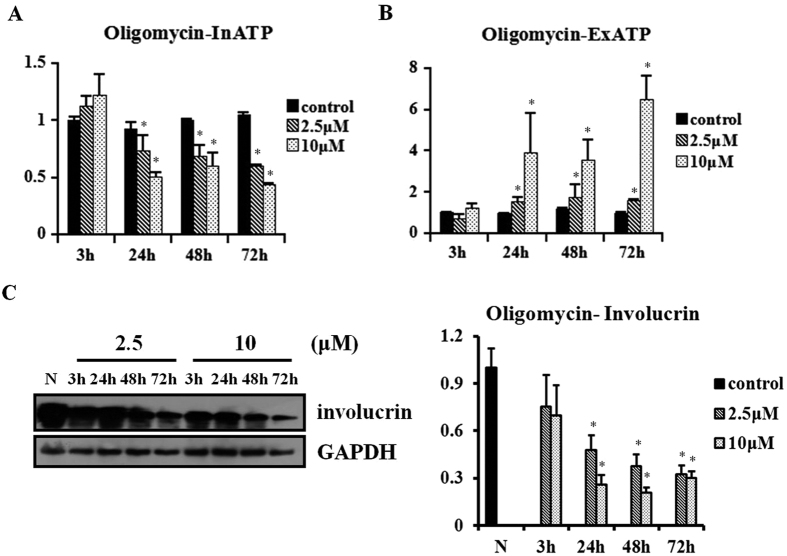
The F1F0-ATP synthase inhibitor oligomycin inhibits InATP synthesis and involucrin expression in HaCaT cells. HaCaT cells under normal culture conditions were exposed to oligomycin (2.5 and 10 μM) at 12 h after cell seeding, for 3, 24, 48, and 72 h. The relative content of ExATP and InATP was analyzed. **(A,B)** InATP content was decreased under treatment of oligomycin at 24, 48, and 72 h, while ExATP content was increased. Bars represent the average values from three independent experiments performed in triplicate, shown as mean ± SEM (*P < 0.05). **(C)** Involucrin expression in HaCaT cells after being exposed to oligomycin (2.5 and 10 μM) was analyzed by western blot. Both doses of oligomycin for 24, 48, and 72 h inhibited involucrin expression. The results are shown as the mean ± SEM of three independent experiments performed in duplicate (*P < 0.05).

Cells were exposed to oligomycin (2.5 and 10 μM) 12 h after cell seeding. Then, InATP and ExATP were measured at 3, 24, 48, and 72 h after exposure. InATP of HaCaT cells at 24, 48, and 72 h after exposure to oligomycin (2.5 and 10 μM) was significantly decreased compared with controls (Fig. 2A). No significant difference was seen in the proliferation inhibition among these three groups. ExATP was significantly increased at 24, 48, and 72 h after exposure to oligomycin (P < 0.05) (Fig. 2B).

Involucrin expression of HaCaT cells was then analyzed at 3, 24, 48, and 72 h after exposure to oligomycin (2.5 and 10 μM). There was a significant decrease of involucrin expression after exposure to both doses of oligomycin for 24, 48, and 72 h (all P < 0.05) (Fig. 2C).

### F1F0-ATP synthase inhibitor piceatannol decreases ExATP, but has no effect on involucrin expression

**Figure 3:**
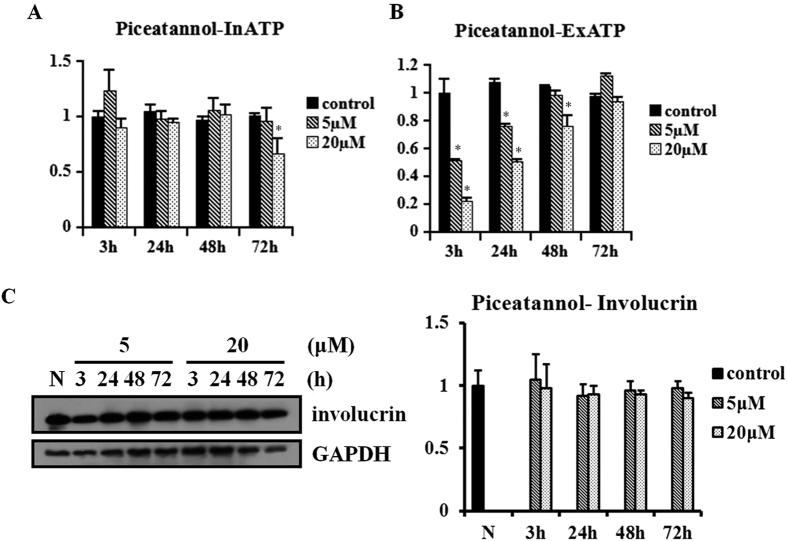
The F1F0-ATP synthase inhibitor piceatannol inhibits ExATP synthesis but has no effect on involucrin expression in HaCaT cells. HaCaT cells under normal culture conditions were exposed to piceatannol (5 and 20 μM) at 12 h after cell seeding for 3, 24, 48, and 72 h. The relative content of ExATP and InATP was analyzed. **(A,B)** ExATP but not InATP content was decreased under piceatannol at 3, 24, and 48 h (20 μM). Bars represent the average value from three independent experiments performed in triplicate, and shown as mean ± SEM (*P < 0.05). **(C)** Piceatannol at different concentrations and exposure times has no effect on involucrin expression, analyzed by western blot. The results are shown as the mean ± SEM of three independent experiments performed in duplicate.

The doses of piceatannol used in the present study were based on previous studies^24^,^25^. Cells were exposed to piceatannol (5 and 20 μM) at 12 h after cell seeding. Then, InATP and ExATP were measured at 3, 24, 48, and 72 h after exposure. After exposure to piceatannol for 3, 24 (5 and 20 μM), and 48 h (20 μM), ExATP content was significantly decreased compared with controls (all P < 0.05). However, only the group exposed to 20 μM piceatannol for 72 h showed a decrease of InATP (P < 0.05) (Fig. 3A,B).

Involucrin expression of the cells was then analyzed at 3, 24, 48, and 72 h after exposure. There was no change of involucrin expression in HaCaT cells after exposure to piceatannol (Fig. 3C).

### ATP5B mRNA is induced in the HaCaT confluence-dependent differentiation model

**Figure 4:**
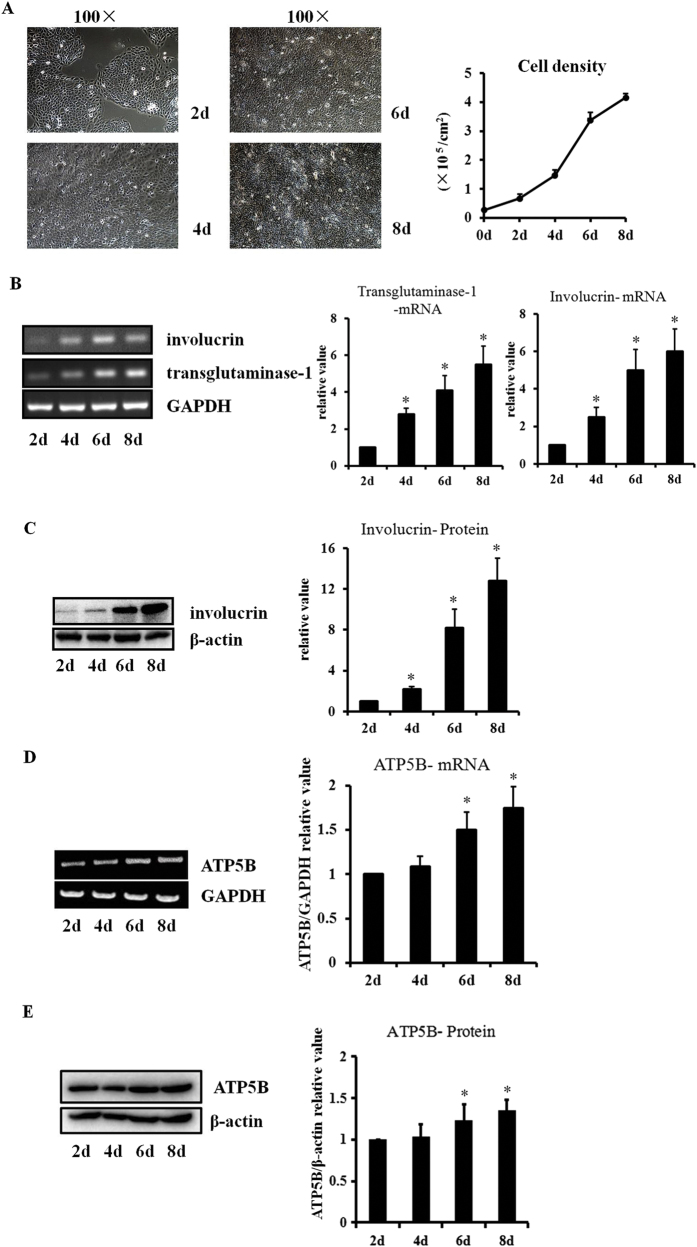
ATP5B is upregulated in the HaCaT confluence-dependent differentiation model. The HaCaT differentiation model was established by seeding cells at day 0 and culturing them for 8 days. **(A)** Cell densities are shown as mean ± SEM, according to cell counting performed with triplicate. **(B,C)** Involucrin and transglutaminase-1 mRNA, as well as involucrin protein were increased with time, accordingly to HaCaT differentiation status (*P < 0.05). **(D,E)** ATP5B mRNA and protein were significantly induced at days 6 and 8 compared with day 2 (*P < 0.05). Bars represent the average values from three independent experiments performed in duplicate, and shown as mean ± SEM.

In order to further investigate the relationship between F1F0-ATP synthase and HaCaT differentiation, a HaCaT confluence-dependent differentiation model was established. At day 0, 1.2 × 10^5^ cells were seeded into 12-well plates, and grown for 8 days. Cells reached 100% confluence on day 4, and the growth rate slowed down in the last 2 days (Fig. 4A).

Involucrin and transglutaminase-1 at 2, 4, 6, and 8 days were analyzed to evaluate and confirm cell differentiation status. The mRNA levels of involucrin and transglutaminase-1 were significantly higher at 4, 6, and 8 days in the confluence model compared with day 2 (P < 0.05) (Fig. 4B). The protein levels of involucrin were significantly upregulated at days 6 and 8 (P < 0.05) (Fig. 4C). Subsequent testing of ATP5B mRNA and protein showed that ATP5B was significantly induced at days 6 and 8 compared with day 2 (Fig. 4D,E).

### F1F0-ATP synthase inhibitor piceatannol inhibits involucrin expression and InATP in the late stage of HaCaT differentiation

In order to investigate the function of F1F0-ATP synthase in HaCaT differentiation, F1F0-ATP synthase inhibitors were used in HaCaT cells. Pilot experiments revealed that oligomycin inhibited cell growth and confluence, while piceatannol had less effect on these parameters (data not shown). Therefore, only piceatannol 5 μM (but not oligomycin) was used for this part of the study.

**Figure 5:**
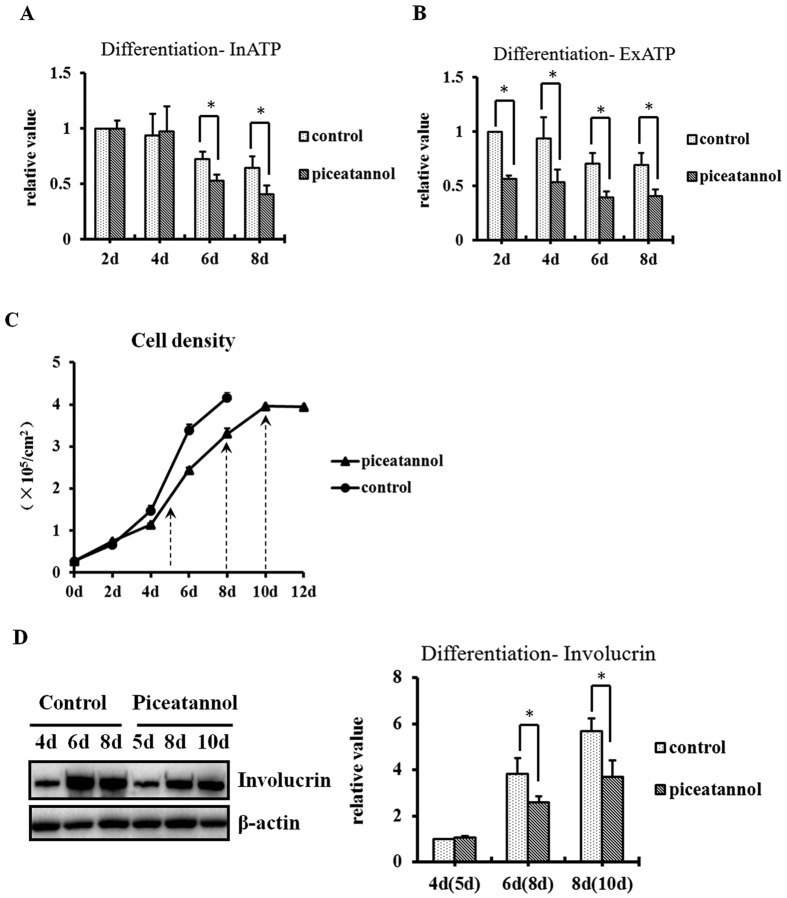
Piceatannol inhibits involucrin expression and intracellular ATP synthesis in the late stage of HaCaT differentiation. **(A,B)** HaCaT cells at days 2, 4, 6, and 8 of the differentiation model were exposed to 5 μM piceatannol for 3 hours before measuring the ATP content. ExATP at 2, 4, 6, and 8 days in the treatment group were all decreased by about 40% after exposure to piceatannol compared with the control groups. InATP at 6 and 8 days in the treatment group were also found to be inhibited by about 30% compared with controls. Results are from three independent experiments performed in triplicate, and shown as mean ± SEM (*P < 0.05). **(C,D)** Piceatannol (5 μM) was applied at 48 h of the differentiation model and was renewed every 24 hours to keep a constant inhibitory effect on ATPase. As piceatannol at working concentration had a slight impact on HaCaT proliferation and cell density, parameters at 5, 8, and 10 days in the piceatannol treatment group (↑) were compared with parameters at 4, 6, and 8 days in the control group. Involucrin expression was analyzed by western blot analysis. Cell densities are shown as mean ± SEM, according to cell counting performed in triplicate. Involucrin expression at days 8 and 10 in the treatment group were found to be decreased by about 30% compared with days 6 and 8 in controls. The results are shown as the mean ± SEM of three independent experiments performed in duplicate (*P < 0.05).

Cells at 2, 4, 6, and 8 days were treated with 5 μM piceatannol for 3 hours and the ATP content was measured. Piceatannol decreased ExATP content of each group by about 40% compared with controls. InATP was also decreased by about 30% after exposure to piceatannol in cells at 6 and 8 days (Fig. 5A,B).

To analyze involucrin expression, because of the limited duration of the effects of piceatannol effect (Fig. 3B), cells were first treated with piceatannol (5 μM) at 48 h of differentiation, and the culture solution with piceatannol (or control) was renewed every 24 hours in order to keep a relatively constant inhibitory effect on ATPase. The growth curves of the two groups were established (Fig. 5C). Considering the differences of proliferation and the confluence-dependent differentiation status, the treatment group was examined at 5, 8, and 10 days, while the control group was examined on days 4, 6, and 8 (Fig. 5C). Involucrin expression was down-regulated by about 30% in the treatment groups at 8 and 10 days (Fig. 5D).

## Discussion

Keratinocyte differentiation is a highly programmed and rigorously controlled process, coordinating cell proliferation and cell death to maintain epidermal homeostasis. Recently, it has been revealed in many reports that mitochondria might play a critical role in the process of keratinocyte differentiation and carcinogenesis, the mechanism for which is under investigation^5^,^26^,^27^. Considering of the predominant functions F1F0-ATP synthase (mitochondrial complex V) plays in mitochondrial respiratory chain and ATP production (specifically InATP in most cells and ExATP in some of them), and the accumulating evidence for the close relation of ATP molecule and cell differentiation, here, we focused on whether and how F1F0-ATP synthase is involved in keratinocyte differentiation. Results showed that ATP5B expression increased with keratinocyte and HaCaT cell differentiation in normal skin, some epidermis hyper-proliferative diseases, SCC, and the HaCaT cell differentiation model. Inhibition of the F1F0-ATP synthase blocked HaCaT cell differentiation, which was associated with a decrease of InATP content, but not with changes of ExATP.

Interestingly, in recent years, proteomic studies indicated a decrease of F1F0-ATP synthase expression in some tumor cell lines and tissues from animal models or patients such as colorectal cancer^28^,^29^, prostate adenocarcinom^30^, and osteosarcoma^31^, and the F1F0-ATP synthase expression correlated with high drug resistance in certain tumors^29^.

To some extent, hyper-proliferative epidermis with impairment of keratinocyte differentiation (such as seen in psoriasis lesion) shares some features with the hypoxic microenvironment and the glycolytic phenotype of tumors^32^. The present study suggests an increase of ATP5B expression with keratinocyte differentiation from the basal cell layer throughout the whole epidermis, which was primarily confirmed in the HaCaT differentiation cell model. In addition, IHC revealed a lower expression of ATP5B in hyper-proliferative epidermis vs. normal tissues, and a lower expression in the poorly differentiated tissue area of SCC compared with well-differentiated areas and near normal peripheral epidermis. These findings are supported by recent oncoproteomics studies in which ATP5B and some other subunits of F1F0-ATP synthase were found to be down-regulated in several kinds of tumor cell lines and tissues such as colorectal cancer, and to be associated with tumor drug-resistance^29^ and oncogenic pathway activation^33^. Few studies made further explorations of the mechanism, but a research group observed down-regulated oxidative phosphorylation enzymes in its proteomic analysis, which was supposed to indicate “metabolic reprogramming” in tumor cells, leading to a shift of glucose metabolism from oxidative phosphorylation to glycolysis^33^,^34^,^35^,^36^.

Interestingly, another part of our study showed that psoriasis epidermis exhibited high expression of pyruvate kinase (PK, one of the key enzymes in glycolysis), glucose transporter-1,4 (GLUT-1,4), and p-Akt. Inhibition of the PI3K/Akt pathway decreased ATP5B and simultaneously increased PK expression in HaCaT cells (unpublished data). Considering of the microenvironment variation throughout keratinocytes differentiation under physiological and pathological conditions, it might be an implication of the metabolic regulatory mechanism of F1F0-ATP synthase in the epidermis. However, further work is necessary to specifically address this issue.

Oligomycin and piceatannol are traditional F1F0-ATP synthase inhibitors, with different targeting sites and acting time, and are widely used in functional studies of the enzyme. At EC50 of ATPase inhibition (4 and 10 μM, respectively), these two small molecular chemicals both displayed suppression effects on HaCaT cell proliferation. Consequently, the HaCaT confluence-dependent differentiation model was slightly delayed by piceatannol (5 μM), while it could not be successfully established under oligomycin and high concentration of piceatannol due to their stronger inhibitory effects. According to suppression on both HaCaT proliferation and differentiation by ATPase inhibitors, it is proposed that, in HaCaT cells, it is not sufficient to create a poor differentiation with hyper-proliferation phenotype by only inhibiting F1F0-ATP synthase.

In the present study, oligomycin mainly inhibited InATP synthesis, while piceatannol mainly inhibited ExATP under normal culturing conditions, which is supported by previous studies^11^,^37^,^38^. However, in the HaCaT differentiation model, piceatannol exhibited a significant inhibitory effect on both InATP and ExATP synthesis in the late period of the model. This was probably due to the change of cell permeability and susceptibility, or altered signaling transduction at that stage. Furthermore, involucrin expression was found to be decreased at the same time as InATP synthesis was inhibited. The consistency of the inhibitory effect on involucrin expression and InATP indicated a possible mechanism of how F1F0-ATP synthase is involved in keratinocyte differentiation, which remains to be confirmed and further elucidated.

ExATP is now widely accepted as a messenger molecule for cell-cell communication, which plays important roles in cell proliferation, differentiation, and apoptosis in various cells including keratinocytes by interacting with specific ATP(ADP) binding receptors (purinergic receptor family)^39^. In the present study, there was no change in involucrin expression while ExATP content was decreased by piceatannol, or increased with oligomycin treatment. Furthermore, supplement of ATP-γ-S to mimic ExATP did not affect involucrin expression either (data not shown). As purinergic receptor family expression was not covered in the present study, expression regulation of purinergic receptors but not the above changes of ExATP content could lead to a constant regulation on HaCaT differentiation programming under physiological conditions.

The present study is not without limitations. In addition to the limitations that are inherent to cell models, we could not use oligomycin in the confluence-dependent HaCaT cell model because of its too important inhibitory effects on the cells. In addition, only a few proteins were tested. Further study with a more comprehensive panel of proteins is necessary to better understand the role of F1F0-ATP synthase in keratinocyte proliferation and skin lesions.

## Conclusion

The present study strongly suggests a possible relationship between F1F0-ATP synthase, InATP, and keratinocyte differentiation. It also provides new insights into the mechanism by which energy metabolism possibly regulates cell biological behaviors like differentiation and proliferation. Future studies are needed to further elucidate the mechanisms in details, and how it is being regulated during keratinocyte differentiation, as well as the signaling network of the InATP.

## Materials and Methods

### Cell culture

Primary keratinocytes have a finite life span, being susceptible to culture conditions and environmental factors. The spontaneously immortalized HaCaT cell line has been a widely used keratinocyte model due to its ease of propagation and a near-normal phenotype^40^. The confluence-dependent HaCaT differentiation model is commonly used in research on keratinocytes differentiation^25^. In this model, after reaching 100% confluence, HaCaT cells move on sequentially to 3–5 mitotic cell cycles, growth arrest, and finally terminal differentiation^41^. Differentiation biomarkers such as involucrin are induced during the process, which is similar to keratinocyte differentiation *in vitro*.

The human immortalized epidermal cell line HaCaT was maintained in culture locally in RPMI1640 medium (Thermo Scientific, MA, USA) supplemented with 10% fetal bovine serum (FBS), 100 U/mL penicillin G and 100 mg/L streptomycin in a humidified 5% CO_2_ atmosphere at 37 °C.

### Cell counting

Cells were washed with PBS and digested with 0.25% trypsin, then suspended and diluted to a set volume with PBS. An automatic cell counter system (Bio-Rad, Hercules, CA, USA) was used for cell counting, in triplicates.

### Immunohistochemistry

All the normal and skin tissue lesions used for immunohistochemistry (IHC) analysis were obtained from patients at the Department of Dermatology, Xiangya Hospital, Central South University, from Jan 2013 to Mar 2014. Tissues were preserved by formalin fixation and paraffin embedding. The tissue samples included nine normal skin tissues, six chronic dermatitis, five prurigo nodularis, seven keratosis seborrheic, nine verruca vulgaris, 25 psoriasis, five keratoacanthoma, and nine squamous cell cancer (SCC) tissues. Paraffin sections were dried and rehydrated, and endogenous peroxidase was blocked by incubation with 0.3% hydrogen peroxide. Antigen activity was retrieved by treating tissue samples with pepsin (Zhongshan, Beijing, China) at 37 °C for 20 min (this step was omitted in K10 analysis) prior to incubation with primary antibody diluted at 1:100 (or 1:50) for ATP5B (Abcam, Cambridge, UK), and 1:1000 for K10 (Abcam, Cambridge, UK) overnight at 4 °C.

The study was approved by the ethics committees of the Xiangya Hospital, Central South University. All patients provided a written, informed consent for tissue banking. The study was conducted in accordance with the Declaration of Helsinki, the International Conference on Harmonization/Good Clinical Practice, applicable regulatory requirements, and Astra Zeneca’s policy on bioethics.

A two-step plus Poly-HRP anti-mouse IgG detection system (Zhongshan, Beijing, China) with diaminobenzidine chromogen (DAB, Zhongshan, Beijing, China) was used for visualization. A humidified box was used for all incubations.

### Preparation of whole cell extracts and western blot

In order to examine the expression of involucrin in the HaCaT cell line, cells were washed with PBS, then collected and solubilized in RIPA buffer on ice for 30 min. The lysate was centrifuged at 12,000 rpm for 15 min at 4 °C to remove cellular debris and the supernatant protein concentration was determined by the BCA protein assay (Santa Cruz Biotechnology, Santa Cruz, CA, USA). The proteins were then added to 5 × loading buffer and incubated at 95 °C for 5 min before being subjected to standard western blotting.

The primary antibody against involucrin (Abcam, Cambridge, UK) were diluted at 1:500, ATP5B antibody (Abcam, Cambridge, UK) was diluted at 1:400, β-actin antibody (Sigma, Germany) was diluted at 1:10,000, and GAPDH antibody (Sigma, Germany) was diluted at 1:5000. Immunolabeled proteins were then visualized using a goat anti-mouse antibody linked to horseradish peroxidase (1:10,000, Sigma, Germany) and an enhanced chemiluminescence detection system (Bio-Rad, Hercules, CA, USA). Protein levels in each lane were normalized to that of β-actin or GAPDH.

### RT-PCR

A reverse transcription polymerase chain reaction (RT-PCR) was performed using the primers (Shenggong, Shanghai, China) listed in Table 1 to detect the mRNA levels of ATP5B, involucrin, and transglutaminase-1 in HaCaT cells.

Total RNA was extract using TRIzol (Invitrogen, Carlsbad, CA, USA) and reverse-transcribed with a reverse transcription kit (Fermentas, Burlington, Canada). Each PCR reaction was composed of 1 μL of cDNA, 1 μL of primers, 10 μL of 2 × Taqmix (Toyobo, Japan), and 8 μL of DEPC water. The cycling variables were: 4-min at 94 °C; then 34 cycles at 94 °C for 30 seconds, 56 °C or 59 °C for 30 minutes (depending on the used primers), and 72 °C for 30 seconds; final extension was at 72 °C for 7 minutes. PCR products (1 μL) were electrophoresed on 1.5% agarose gels. The grey scale ratio of the target genes with β-actin was calculated for further analysis.

### Measurement of InATP and ExATP

The aluminometric ATP assay system (ATP assay kit, Beyotime Institute of Biotechnology, Haimen, China) was used to measure the InATP and ExATP content in HaCaT cells, based on. In brief, for measurement of the InATP content, cells in each well were washed with PBS, and solubilized with 200 μL of lysis buffer at 0 °C for 30 min. The lysate was centrifuged at 12,000 rpm for 15 min at 4 °C and kept at 0–4 °C for further ATP measuring and BCA protein assay. For measurement of the ExATP content, cells (in 12-well culture plate) were washed with preheated PBS, and incubated with 200 μL of PBS containing 200 nM ADP at 37 °C for 15–30 seconds; then, the ADP solution was transferred in each well to a tube kept at 0–4 °C for ATP measuring. The remaining cells were subjected to BCA protein assay (same as above).

Samples and ATP standard solutions were mixed with the ATP assay working solution in a 96-well plate and immediately placed in a multifunctional microplate reader (DTX 880; Beckman Coulter, Brea, CA, USA). Light emission was recorded to determine the ATP concentration. For each well, two replicates were used to gain an average value. The final ATP content of each well was standardized by the corresponding total protein concentration.

### F1F0-ATP synthase inhibitors and ATP

The inhibitors of F1F0-ATP synthase, oligomycin and piceatannol, were purchased from Aladdin Reagent Co. Ltd. (Shanghai, China). ADP used to pretreat the cells for ATP measurement was purchased from Sigma-Aldrich (St Louis, MO, USA). ATP-γ-S, as an analogue of ATP, was purchased from Sigma-Aldrich (St Louis, MO, USA).

### Statistical analysis

SPSS 13.0 (SPSS Inc., Chicago, IL, USA) was used for statistical analysis. Data were analyzed using the Student’s t test. A P-value < 0.05 was considered statistically significant.

## Additional Information

**How to cite this article:** Xiaoyun, X. *et al*. Possible Involvement of F1F0-ATP synthase and Intracellular ATP in Keratinocyte Differentiation in normal skin and skin lesions. *Sci. Rep.* **7**, 42672; doi: 10.1038/srep42672 (2017).

**Publisher's note:** Springer Nature remains neutral with regard to jurisdictional claims in published maps and institutional affiliations.

## Figures and Tables

**Table 1:** Primer sequences.

Target	Sequences
*ATP5B*	F 5′- GTTGGGGTTTGTGGGTCGGGTG
	R 5′-TTTGGCGAAGGAGATGTTTGCG
*IVL (involucrin)*	F 5′-TGCCTGAGCAAGAATGTGAG
	R 5′-AGCTGCTGATCCCTTTGTGT
*TGM1 (transglutaminase-1)*	F 5′-TCTGATGGTCTCTGTGATGCTGAT
	R 5′-TCCACTTCCTTCTTGGTCTCCTT
*Actb (β-actin)*	F 5′-ACTCTTCCAGCCTTCCTTCC
	R 5′-GTACTTGCGCTCAGGAGGAG
